# Supplementary material for: Causal Relationship between Aging and Anorexia Nervosa: A White-Matter-Microstructure-Mediated Mendelian Randomization Analysis
Source: Biomedicines. 2024 Aug 16;12(8):1874. doi: 10.3390/biomedicines12081874 (PMC11351342; doi:10.3390/biomedicines12081874)
Supplement: Supplementary file 1 [file biomedicines-12-01874-s001.zip › Supplementary Figure/Figure S4/Singe SNP plot of MR analysis for TL to ubm-b-1908.pdf]

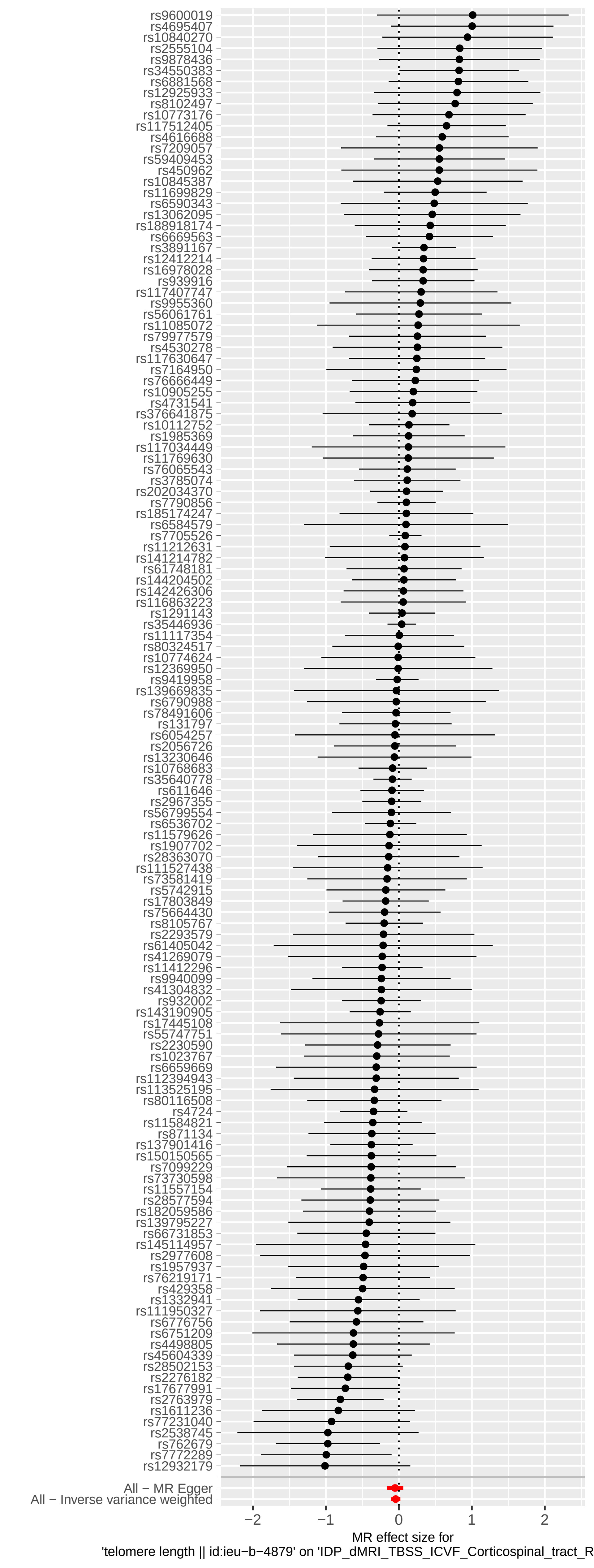
rs9600019
rs4695407
rs10840270
rs2555104
rs9878436
rs34550383
rs6881568
rs12925933
rs8102497
rs10773176
rs117512405
rs4616688
rs7209057
rs59409453
rs450962
rs10845387
rs11699829
rs6590343
rs13062095
rs188918174
rs6669563
rs3891167
rs12412214
rs16978028
rs939916
rs117407747
rs9955360
rs56061761
rs11085072
rs79977579
rs4530278
rs117630647
rs7164950
rs76666449
rs10905255
rs4731541
rs376641875
rs10112752
rs1985369
rs117034449
rs11769630
rs76065543
rs3785074
rs202034370
rs7790856
rs185174247
rs6584579
rs7705526
rs11212631
rs141214782
rs61748181
rs144204502
rs142426306
rs116863223
rs1291143
rs35446936
rs11117354
rs80324517
rs10774624
rs12369950
rs9419958
rs139669835
rs6790988
rs78491606
rs131797
rs6054257
rs2056726
rs13230646
rs10768683
rs35640778
rs611646
rs2967355
rs56799554
rs6536702
rs11579626
rs1907702
rs28363070
rs111527438
rs73581419
rs5742915
rs17803849
rs75664430
rs8105767
rs2293579
rs61405042
rs41269079
rs11412296
rs9940099
rs41304832
rs932002
rs143190905
rs17445108
rs55747751
rs2230590
rs1023767
rs6659669
rs112394943
rs113525195
rs80116508
rs4724
rs11584821
rs871134
rs137901416
rs150150565
rs7099229
rs73730598
rs11557154
rs28577594
rs182059586
rs139795227
rs66731853
rs145114957
rs2977608
rs1957937
rs76219171
rs429358
rs1332941
rs111950327
rs6776756
rs6751209
rs4498805
rs45604339
rs28502153
rs2276182
rs17677991
rs2763979
rs1611236
rs77231040
rs2538745
rs762679
rs7772289
rs12932179
All − MR Egger
All − Inverse variance weighted
−2
−1
0
1
2
MR effect size for
'telomere length || id:ieu−b−4879' on 'IDP_dMRI_TBSS_ICVF_Corticospinal_tract_R
